# Identification for heavy metals exposure on osteoarthritis among aging people and Machine learning for prediction: A study based on NHANES 2011-2020

**DOI:** 10.3389/fpubh.2022.906774

**Published:** 2022-08-01

**Authors:** Fang Xia, Qingwen Li, Xin Luo, Jinyi Wu

**Affiliations:** Department of Public Health, Wuhan Fourth Hospital, Wuhan, China

**Keywords:** osteoarthritis, metal elements, XGBoost, NHANES, risk factors, aging people

## Abstract

**Objective:**

Heavy metals are present in many environmental pollutants, and have cumulative effects on the human body through water or food, which can lead to several diseases, including osteoarthritis (OA). In this research, we aimed to explore the association between heavy metals and OA.

**Methods:**

We extracted 18 variables including age, gender, race, education level, marital status, smoking status, body mass index (BMI), physical activity, diabetes mellitus, hypertension, poverty level index (PLI), Lead (Pb), cadmium (Cd), mercury (Hg), selenium (Se), manganese (Mn), and OA status from National Health and Nutrition Examination Survey (NHANES) 2011-2020 datasets.

**Results:**

In the baseline data, the *t* test and Chi-square test were conducted. For heavy metals, quartile description and limit of detection (LOD) were adopted. To analyze the association between heavy metals and OA among elderly subjects, multivariable logistic regression was conducted and subgroup logistic by gender was also carried out. Furthermore, to make predictions based on heavy metals for OA, we compared eight machine learning algorithms, and XGBoost (AUC of 0.8, accuracy value of 0.773, and kappa value of 0.358) was the best machine learning model for prediction. For interactive use, a shiny application was made (https://alanwu.shinyapps.io/NHANES-OA/).

**Conclusion:**

The overall and gender subgroup logistic regressions all showed that Pb and Cd promoted the prevalence of OA while Mn could be a protective factor of OA prevalence among the elderly population of the United States. Furthermore, XGBoost model was trained for OA prediction.

## Introduction

Osteoarthritis (OA) was a degenerative joint disease characterized by loss of articular cartilage and progressive degeneration, which was the most common chronic joint disease and the main cause of functional disability in the elderly (1, 2). Among the 291 diseases in the 2010 global disease burden research, knee and hip osteoarthritis was ranked as the 11th highest cause of global disability and the 38th highest cause of disability-adjusted life years (DALYs). The age-standardized prevalence of knee and hip osteoarthritis were 3.8 and 0.85%, respectively. Between 1990 and 2010, the DALYs of knee and hip OA increased from 10.5 million (0.24% of the total DALYs) to 17.1 million (0.69% of the total DALYs) (3, 4). Xu and Wu analyzed an NHANES dataset from 2005 to 2018, and there was an increasing trend in the age-adjusted prevalence of OA in both men and women in the USA (5). Price et al. reported that more than 95% of all knee replacements were done for osteoarthritis. There were more than 100,000 knee replacements in the UK and 700,000 knee replacements in the USA each year. The number of osteoarthritis cases was increasing as predicted despite periods of economic downturn (6).

The pathogenesis of OA was characterized by cartilage erosion, abnormal bone remodeling, and chronic low-grade synovitis. The production of intra-articular proinflammatory cytokines led to the production of reactive oxygen species, such as peroxides, hydroxylated free radicals, and nitric oxide, which was accompanied by the down-regulation of antioxidants (3, 4). The oxidative stress led to the upregulation of catabolic enzymes, the degradation of extracellular matrix, the reduction of matrix synthesis, joint inflammation, chondrocyte death, and aging, resulting in the overall progress of the disease (7).

Since oxidative stress had association with pathogenesis of OA, heavy metals should be analyzed for it could induce oxidative stress cellular response. It was reported that with the increase in obesity, knee injury, and life expectancy, the prevalence of OA in developing countries was rising. Previous studies also found that there were synergistic or antagonistic effects between chemical elements in the bone tissue samples of OA patients, and their exposure to environmental factors, such as smoking, diet, exercise, and nutrition supplements (8). In addition, some reports found that the level of anterior cruciate ligament Hg was significantly increased in female patients under 65 years old, with spinal degenerative diseases (2, 9). However, limited information was known about the correlation between OA and heavy metals, which was necessary for further exploration. The exploration of heavy metals associated with OA was imperative since people would experience an accumulation of heavy metals in some working circumstances and OA was a threat to people, especially the elderly. The association analysis and prediction for OA could help people prevent OA and reduce risk factor exposure.

## Methods

### Dataset

NHANES was a program of studies designed to assess the health and nutritional status of adults and children in the United States. All the data can be found on the website of the American Centers for Disease Control and Prevention (https://www.cdc.gov/nchs/nhanes). In this research, the National Health and Nutrition Examination Survey (NHANES) in 2011-2020 containing demographic, disease history, economics, and five heavy metals was used, since only five heavy metals in blood were reported in the large-scale population during 2011-2022.

To analyze the association between OA and 5 heavy metals (Pb, Cd, Hg, Se, and Mn) among aging people, we first analyzed the LOD, quartile value, and geometric mean (GM) of each metal. Then we conducted a multivariable logistic regression to find out significant metal for OA (adjusted for age, gender, race/ethnicity, education level, marital status, smoking, BMI, physical activity, diabetes mellitus, hypertension, and PLI). Moreover, gender subgroup logistic analyses adjusted for the above covariates were also carried out.

The demographic data included several OA-related factors containing age, gender, race/ethnicity, education level, marital status, smoking, BMI, physical activity, diabetes mellitus, hypertension, and PLI. The heavy metals were Lead (Pb), cadmium (Cd), mercury (Hg), selenium (Se), and manganese (Mn). Since heavy metals in blood could better reflect heavy metal accumulation, we chose blood heavy metals. The outcome of OA had two statuses, OA and no OA.

**Figure 1:**
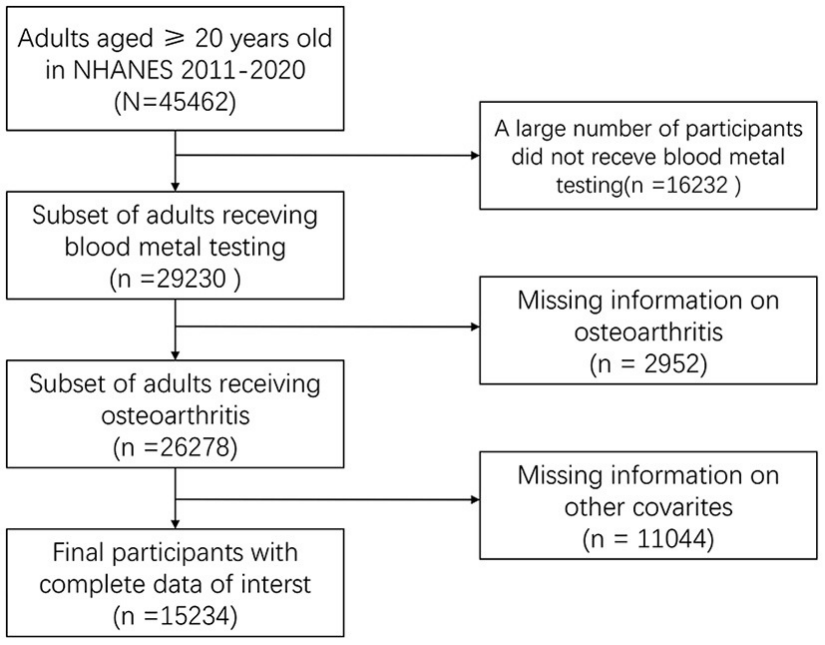
Flowchart of dataset combination.

To screen data, we searched for the OA-related data in NHANES 2011-2020. In the raw data, there were 45,462 participants, of which 29,230 respondents had lab data and 26,278 samples had OA status data. In the end, 15,234 participants with demographic, disease history, economic, five heavy metals, and OA status were included (Figure 1).

### Evaluation of osteoarthritis

OA status was obtained by conducting a questionnaire survey. Participants were asked 'Did a doctor or other healthcare professional ever tell you that you have arthritis?' If the answer to the first question was “yes,” a follow-up question “What type of arthritis was it?” would be asked. Participants were classified as with or without OA based on their different answers to these two questions. A previous study showed that consistency between self-reported OA and clinically confirmed OA reached 85% (10).

### Assessment of heavy metals

Heavy metals in blood were measured directly in Lead (Pb), cadmium (Cd), total mercury (Hg), manganese (Mn), and selenium (Se) in whole blood samples using mass spectrometry after a simple dilution sample preparation step. During the dilution phase of the sample, a small volume of whole blood was extracted from a larger sample of whole blood patients after the entire sample has been mixed to create a uniform distribution of cellular components. Dilution of blood during sample preparation before analysis consisted of a simple dilution of 1 sample part + 1 water part + 48 diluent parts. Liquid samples were introduced into the mass spectrometer through the inductively coupled plasma ionization source.

### Covariates

Information on demography and lifestyle factors was collected through questionnaires. Baseline data were age (continuous), gender (male, female), race/ethnicity (non-Hispanic white, non-Hispanic black, Mexican American, others), education (high school, high school or equivalent and high school), marital status (married/cohabiting, widowed/divorced/separated and never married), smoking status (current, never), physical activity (Yes and No) and poverty level index (PLI) (≤ 1, 1 < – ≤ 3, >3). The medical examination was carried out in mobile centers. The body mass index (BMI, kg/m^2^) was classified as normal weight <25, overweight 25– <30, and obesity ≥ 30. Diabetes was defined as reaching a fasting glucose level ≥ 126 mg/dl or reporting a previous diagnosis (11). Hypertension was defined as resting blood pressure (BP) persistent at 140/90 mmHg or reporting a previous diagnosis (12).

### Statistical analysis

In the baseline data analysis, the Cochran-Mantel-Haenszel test and *t* test were used to analyze the difference in demographic data in the two OA groups. Subsequently, we used quartile analysis and logistic regression to identify meaningful heavy metals associated with OA. The multiple variables logistic regression could be used to find out the harmful metal and protective metal for OA. A Mendelian randomization study analyzed causal associations between serum nutritional factors and OA in men and women. A causal effect of serum selenium levels on OA both in men and women was also observed (13). In this research, we conducted a gender subgroup analysis of heavy metals for OA. Furthermore, some systematic review and meta-analysis revealed that there was an inverse association between cigarette smoking and the risk of knee OA, so we conducted a smoking subgroup analysis.

After the above analyses, we compared eight machine learning algorithms based on area under curve (AUC), accuracy, and kappa value. The best machine learning model would be used to make the prediction for OA based on multiple variables including demographic, lifestyle, disease, and heavy metal data. To validate the prediction effects, cross-validation was conducted, in which 80% of the data was used to train the model and 20% of the data was used to make the prediction. Then, the prediction value would be evaluated with the remaining 20% original data. The confusion matrix and ROC curve would be used to evaluate the prediction effects. To apply the XGBoost prediction, an online shiny app was developed using R shiny package. All the analyses were conducted in R software 4.1.2 (The R Foundation for Statistical Computing, USA). Two-sided *P* < 0.05 was considered statistically significant.

### Machine learning component

Since there were eight machine learning algorithms commonly used for prediction (14, 15), we compared the eight different machine learning algorithms including Extreme Gradient Boosting (XGBoost), Decision Tree (DT), Support Vector Machine (SVM), Multivariate Adaptive Regression Splines (MARS), Artificial Neural Networks (ANN), Boosted Trees (BT), Random Forest (RF), and K-Nearest Neighbors (KNN). In the comparison of the eight algorithms, XGBoost was the best choice (xgbTree package in R was used).

XGBoost was a library optimized to increase distributed gradient and designed to be highly efficient, flexible, and portable. We implemented machine learning algorithms under the Gradient Boosting framework. XGBoost provided a parallel tree augmentation (also known as GBDT, GBM) that solved many data science problems quickly and accurately. The same code worked on major distributed environments (Hadoop, SGE, MPI) and could solve problems over billions of examples. The principle of the XGBoost algorithm could be summarized as follows:

We assumed a training dataset D = {(x_i_, y_i_), i = 1..n} of the size n, where xi = (x_i1_, x_i2_,…, x_I_) denoted an m-dimensional feature vector with the corresponding (output) category y_i_:


$$\begin{array}{l} \hat{Y_{i}}=\overset{k}{\underset{k=1}{\sum }}f_{k}\left(x_{i}\right),f_{k}\in F \end{array}$$

where K represented the number of trees, f_k_ (x_i_) represented the score that was associated with the model's k-th tree, and F denoted the space of scoring functions available for all boosting trees.

Different from another tree-based algorithm GBDT (gradient boosting decision tree), XGBoost used the second-order Taylor expansion to approximate the loss function, and mainly avoided the overfitting problem by adding a regularization term to the objective function (16).

### XGBoost model fitting and validation

Firstly, we divided the original data into training data and testing data according to an 8:2 ratio. Training data was used to train the XGBoost model and testing data was used to verify the model. We validated each algorithm 10 times *via* cross-validation.

In the process of adapting the model, we set parameters including the number of trees increasing, maximum tree depth, age, fall rate, skip of drop, col sample by tree, minimum child weight, sub-sample and range. The parameters wre as follows: nrounds = c (1, 13, 17), max_depth=2, eta=0.1, rate_drop=0.10, skip_drop=0.10, colsample_bytree=0.90, min_child_weight=2, subsample=0.75, gamma=0.10.

To select the characteristics and evaluate the adaptation of the model, diagrams of variable importance and ROC curve were conducted. In addition, the confusion matrix was applied to evaluate the XGBoost model prediction and several indicators were used including sensitivity, specificity, positive prediction value, negative prediction value, Kappa value, and accuracy.

## Results

### Characteristics of participants

A total of 15,234 people with mean age 49.6 were included in this study, including 4,214 with OA and 11,020 without OA (Table 1). In the analysis of baseline data, it could be seen that the average age of patients with OA was significantly higher than that of patients without OA. In patients with OA, the proportion of women was higher than that of non-OA patients. There were differences in demographic data such as race, education level, and marital status between the OA group and no OA group. There were also significant differences in individual BMI, smoking, and exercise between the OA group and no OA group. In comparison with the history of disease, hypertension, and diabetes, there was difference between OA and non-OA. Finally, in the comparison of family income and PLI index, there were also differences between the two groups. Concerning heavy metals, the detectable percentage of blood levels of metal ≥ LOD, GM (95% CI) and quartiles of concentrations of the five blood metals were listed in Table 2.

**Table 1:** Characteristics of participants by osteoarthritis status in American aging people from NHANES 2011-2020.

**Characteristics**	**OA** **(*N* = 4,214)**	**No arthritis** **(*N* = 11,020)**	**t or Chi-square value**
**Age** **(years, mean** **±SD)**	61.53 ± 13.71	45.04 ± 16.63	62.42^*^
**Gender**, ***n*** **(%)**			
Male	1,697 (40.27%)	5,688 (51.61%)	156.62^*^
Female	2,517 (59.73%)	5,332 (48.39%)	
**Race**, ***n*** **(%)**			
Mexican American	347 (8.23%)	1,420 (12.88%)	357.67^*^
Other hispanic	396 (9.39%)	1,160 (10.52%)	
Non-hispanic white	2,044 (48.5%)	3,894 (35.33%)	
Non-hispanic black	1,043 (24.75%)	2,550 (23.13%)	
Other race	384 (9.13%)	1,996 (18.14%)	
**Education**, ***n*** **(%)**			
<9th grade	432 (10.25%)	792 (7.18%)	154.63^*^
9–11th grade	535 (12.69%)	1,223 (11.09%)	
High school graduate or equivalent	1,012 (24.01%)	2,433 (22.07%)	
Some college or AA degree	1,424 (33.79%)	3,451 (31.31%)	
College graduate or above	808 (19.19%)	3,118 (28.32%)	
Refused	0 (0%)	1 (0.01%)	
Don't know	3 (0.07%)	2 (0.02%)	
**Marital status**, ***n*** **(%)**			
Married/living with partner	1,101 (26.12%)	2,607 (23.65%)	6,308^*^
Widowed/divorced/separated	657 (15.59%)	768 (6.96%)	
Never married	207 (4.91%)	962 (8.72%)	
Refused	1 (0.02%)	1 (0.01%)	
Missing	2,248 (53.36%)	6,682 (60.66%)	
**BMI**, ***n*** **(%)**			
Normal (25 < )	788 (18.69%)	3,473 (31.52%)	338.28^*^
Overweight (25 ≤ BMI <30)	1,291 (30.63%)	3,550 (32.21%)	
Obesity (≥30)	2,135 (50.68%)	3,997 (36.27%)	
**Smoking status**, ***n*** **(%)**			
Yes	2,219 (52.65%)	4,312 (39.14%)	229.63^*^
No	1,995 (47.35%)	6,701 (60.8%)	
Refused	0 (0%)	2 (0.02%)	
Don't know	0 (0%)	5 (0.04%)	
**Physical activity**, ***n*** **(%)**			
Yes	757 (17.96%)	2,435 (22.09%)	38.00^*^
No	3,453 (81.95%)	8,584 (77.9%)	
Don't know	4 (0.09%)	1 (0.01%)	
**Diabetes**, ***n*** **(%)**			
Yes	1,035 (24.56%)	1,087 (9.88%)	618.19^*^
No	3,005 (71.4%)	9,695 (87.97%)	
Borderline	172 (4.08%)	234 (2.12%)	
Don't know	2 (0.05%)	4 (0.03%)	
**Hypertension**, ***n*** **(%)**			
Yes	2,576 (61.14%)	3,058 (27.76%)	1,462^*^
No	1,632 (38.72%)	7,954 (72.17%)	
Don't know	6 (0.14%)	8 (0.07%)	
**PLI (poverty level index)**			
1 ≤	1,041 (24.7%)	2,658 (24.11%)	13.50^*^
1 < – ≤ 3	1,940 (46.05%)	4,803 (43.6%)	
>3	1,233 (29.25%)	3,559 (32.29%)	

* *P <0.05, OA, Osteoarthritis*.

**Table 2:** Blood levels of heavy metals (ug/L) by osteoarthritis status in US aging people from NHANES 2011–2020.

≥**LOD(%)**		**OA (*****N*** = **4,214)**				
		**GM (95%CI)**	**Quartile 1**	**Quartile 2**	**Quartile 3**	**Quartile 4**
Lead	99.98%	11.75 (11.52, 11.99)	<7.7	7.7–11.6	11.6–17.8	>17.8
Cadmium	95.76%	0.38 (0.37, 0.39)	<0.22	0.22–0.36	0.36–0.64	>0.64
Mercury	84.54%	0.78 (0.76, 0.80)	<0.38	0.38–0.73	0.73–1.48	>1.48
Selenium	100.00%	187.58 (186.76, 188.40)	<172.1	172.1–187.2	187.2–204.01	>204.01
Manganese	100.00%	8.88 (8.79, 8.98)	<7.06	7.06–8.83	8.83–11.08	>11.08
		**No arthritis (*****N*** = **11,020)**				
		**GM (95%CI)**	**Quartile 1**	**Quartile 2**	**Quartile 3**	**Quartile 4**
Lead		9.39 (9.26, 9.51)	<5.7	5.7–9.1	9.1–14.8	>14.8
Cadmium		0.32 (0.32, 0.33)	<0.18	0.18–0.3	0.3–0.54	>0.54
Mercury		0.82(0.81, 0.84)	<0.37	0.37–0.74	0.74–1.59	>1.59
Selenium		190.24 (189.77, 190.72)	<175	175–189.94	189.94–205.75	>205.75
Manganese		9.45 (9.38, 9.51)	<7.49	7.49–9.33	9.33–11.75	>11.75

### Associations of blood metal metabolites with OA

According to the analysis of baseline data, we included all demographic data in a multivariate logistic regression to analyze the risk factors leading to OA and its 95% CI (Table 3). Taking the first quartile as a reference, the third quartile of Pb would lead to a higher incidence of OA [OR = 1.02, 95%CI = (1.01, 1.04), *P* = 0.035]. In Cd element analysis, the fourth quartile led to a higher incidence of OA [OR = 1.02, 95%CI= (1.01, 1.04), *P* = 0.035]. The third [OR = 0.97, 95%CI = (0.95, 0.99), *P* = 0.033] and fourth [OR = 0.99, 95%CI = (0.97, 1), *P* = 0.019] quartiles of Mn all played a protective effect, and had a lower OA incidence than first quartile. No correlation was found between Hg or Se and OA.

**Table 3:** Association between blood metals (ug/L) and osteoarthritis in elderly American subject with gender subgroups, from NHANES 2011-2020.

	**Total (*****N*** = **15,234)**		**Male (*****N*** = **7,385)**		**Female (*****N*** = **7,849)**	
	**OR (95%CI)**	***P*-value**	**OR (95%CI)**	***P*-value**	**OR (95%CI)**	***P*-value**
**Pb**						
Quartile 1	1	-	1	-	1	-
Quartile 2	1.02 (0.98, 106)	0.288	1.09 (1.03, 1.16)	0.005^*^	0.98 (0.94, 1.02)	0.327
Quartile 3	1.02(1.01, 1.04)	0.035^*^	1.03 (1, 1.06)	0.024^*^	1.01 (0.98, 1.03)	0.638
Quartile 4	1 (0.99, 1.01)	0.139	1 (0.99, 1.01)	0.071	1.01 (0.99, 1.01)	0.795
**Cd**						
Quartile 1	1	-	1	-	1	-
Quartile 2	1.32 (0.31, 5.29)	0.699	1.4 (0.94, 1.8)	0.141	0.74 (0.15, 3.38)	0.698
Quartile 3	1.13 (0.83, 1.51)	0.427	0.97 (0.38, 2.13)	0.944	1.13 (0.82, 1.55)	0.446
Quartile 4	1.14 (1.05, 1.23)	0.001^*^	1.17 (1.04, 1.31)	0.009^*^	1.12 (1, 1.25)	0.043^*^
**Hg**						
Quartile 1	1	-	1	-	1	-
Quartile 2	0.82 (0.63, 1.03)	0.107	0.45 (0.15, 0.87)	0.066	0.92 (0.69, 1.16)	0.539
Quartile 3	1.02(0.94, 1.09)	0.623	0.87(0.69, 1.05)	0.208	1.06(0.97, 1.17)	0.182
Quartile 4	0.99(0.97, 1.01)	0.194	0.98(0.96, 1.01)	0.264	0.99(0.96, 1.02)	0.456
**Se**						
Quartile 1	1	-	1	-	1	-
Quartile 2	0.97 (0.97, 1)	0.344	0.99 (0.99, 1)	0.488	0.99 (0.99, 1)	0.358
Quartile 3	0.98 (0.98, 1)	0.102	0.99 (0.99, 1.01)	0.102	0.99 (0.99, 1.01)	0.317
Quartile 4	0.99 (0.99, 1)	0.341	0.99 (0.99, 1)	0.162	0.99 (0.98, 1.01)	0.684
**Mn**						
Quartile 1	1	-	1	-	1	-
Quartile 2	0.96 (0.92, 1.01)	0.097	0.99 (0.93, 1.05)	0.641	0.94 (0.88, 1.01)	0.06
Quartile 3	0.97 (0.95, 0.99)	0.033^*^	0.97 (0.94, 1.01)	0.143	0.97 (0.94, 1.01)	0.157
Quartile 4	0.99 (0.97, 1)	0.019^*^	0.98 (0.96, 1.01)	0.064	0.99 (0.98, 1.01)	0.389

* *P < 0.05*.

### Subgroup analysis in gender

In the gender subgroup analysis (Table 3), the incidence of male OA influenced by Pb was higher in the second quartile [OR = 1.09, 95%CI = (1.03, 1.16), *P* = 0.005] and third quartile [OR = 1.03, 95%CI = (1, 1.06), *P* = 0.024] than in the first quartile, which was consistent with the results in the overall logistic regression. The results of Cd in men [OR = 1.17, 95%CI = (1.04, 1.31), *P* = 0.009] and women [OR = 1.12, 95%CI = (1, 1.25), *P* = 0.043] were in line with the overall logistic results. The fourth quartile of men and women all resulted in a higher incidence of OA than the first quartile. In the subgroup analysis of Hg, Se, and Mn, there were no statistically significant risk factors.

### Subgroup analysis in smoking

Concerning Pb, the fourth quartile of the smoking group had an association with OA [OR = 1.01, 95%CI = (1, 1.01), *P* = 0.038] when the third quartil of the total samples had significance in the association [OR = 1.02, 95%CI = (1.01, 1.04), *P* = 0.035], which indicated that smoking might have inverse effects in the correlation between Pb and OA (Table 4). However, there was no significance between Pb and OA in the non-smoking group. In Cd analysis, smoking group had higher [OR = 1.17, 95%CI = (1.07, 1.27), *P* = 0.0003] OR value than total samples [OR = 1.14, 95%CI = (1.05, 1.23), *P* = 0.001], and total samples had much higher OR value than non-smoking group [OR = 0.67, 95%CI = (0.49, 0.91), *P* = 0.01]. These showed that non-smoking might alleviate the association between Cd and OA, while smoking might improve the association. In Mn analysis, both smoking and non-smoking groups had no significance, while total samples' Mn had an association with OA. In the Hg and Se sections, total participants and smoking subgroups had no significance.

**Table 4:** Subgroup analysis of smoking in the association between blood metals (ug/L) and osteoarthritis in American aging people from NHANES 2011-2020.

	**Smoking (*****N*** = **6,531)**		**Non-smoking (*****N*** = **8,696)**	
	**OR (95%CI)**	***P*-value**	**OR (95%CI)**	***P*-value**
**Pb**				
Quartile 1	1	-	1	-
Quartile 2	1.02 (0.97, 1.08)	0.478	1.01 (0.97, 1.06)	0.569
Quartile 3	1.03 (1, 1.05)	0.063	1.01 (0.99, 1.04)	0.375
Quartile 4	1.01 (1, 1.01)	0.038^*^	0.99 (0.99, 1.01)	0.818
**Cd**				
Quartile 1	1	-	1	-
Quartile 2	2.30 (0.35, 1.41)	0.361	0.13 (0.002, 4.11)	0.275
Quartile 3	1.21 (0.88, 1.65)	0.228	0.61 (0.21, 1.59)	0.328
Quartile 4	1.17 (1.07, 1.27)	0.0003^*^	0.67 (0.49, 0.91)	0.01^*^
**Hg**				
Quartile 1	1	-	1	-
Quartile 2	0.64 (0.28, 1.17)	0.215	0.89 (0.67, 1.13)	0.415
Quartile 3	1 (0.83, 1.19)	0.969	1.04 (0.95, 1.14)	0.337
Quartile 4	0.98 (0.95, 1.01)	0.138	0.99 (0.97, 1.02)	0.749
**Se**				
Quartile 1	1	-	1	-
Quartile 2	0.99 (0.99, 1)	0.219	0.99 (0.99, 1)	0.358
Quartile 3	0.99 (0.99, 1.01)	0.115	0.99 (0.99, 1.01)	0.317
Quartile 4	0.99 (0.99, 1)	0.355	0.99 (0.98, 1.01)	0.684
**Mn**				
Quartile 1	1	-	1	-
Quartile 2	0.97 (0.91, 1.02)	0.238	0.96 (0.91, 1.03)	0.258
Quartile 3	0.97 (0.94, 1.01)	0.143	0.98 (0.94, 1.02)	0.229
Quartile 4	0.99 (0.97, 1)	0.107	0.99 (0.97, 1.01)	0.156

* *P < 0.05*.

**Figure 2:**
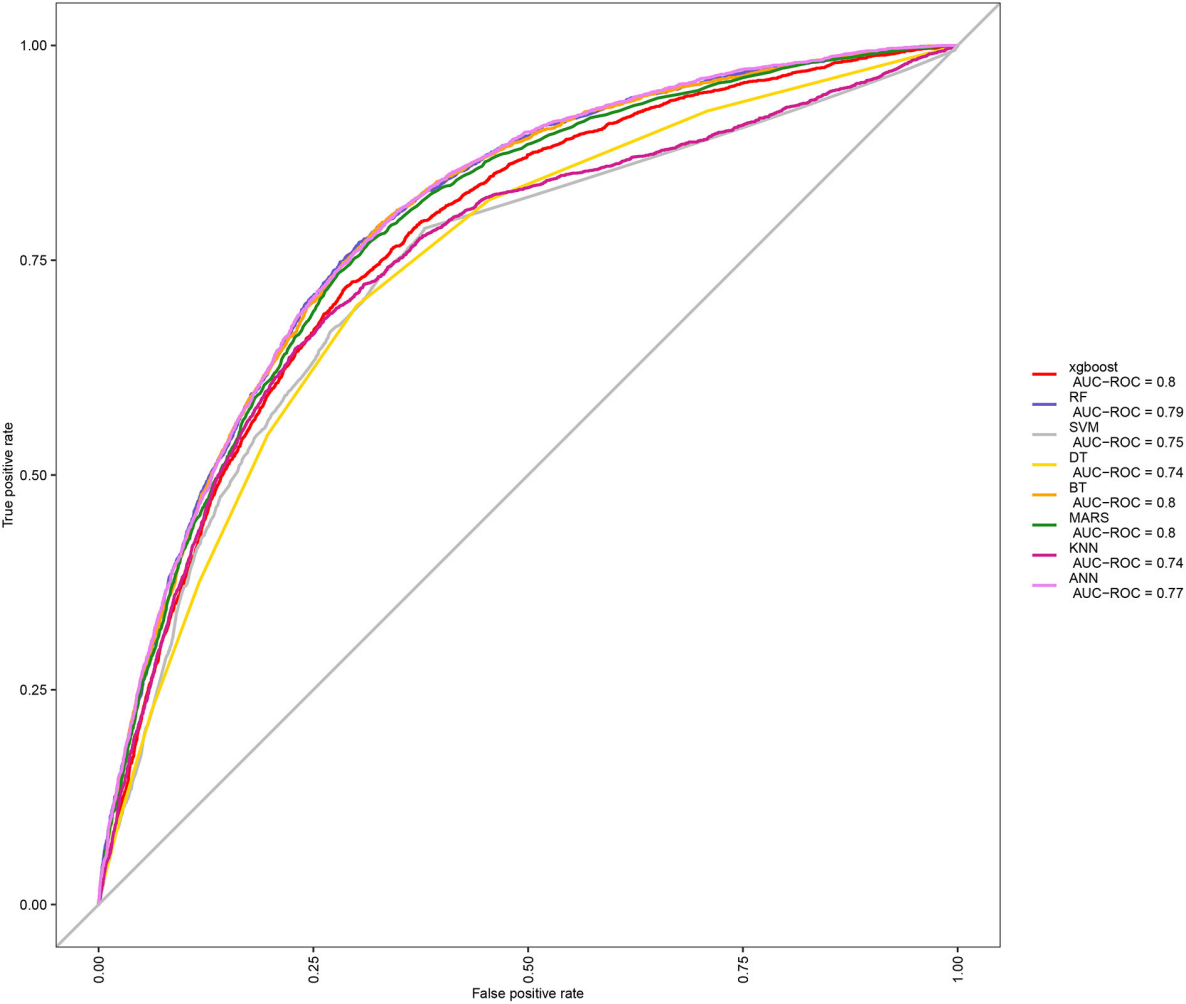
Comparison among eight machine learning algorithms in ROC curve.

After identifications of blood metal risk factors of OA, in order to further apply these associations, we put Pb, Cd, and Mn blood metals and other baseline data into the machine learning model to predict the probability of individual OA. Because there were many machine learning methods, we compared the ROC curves and AUC values among eight machine learning models (Figure 2), including XGBoost, RF, SVM, DT, BT, MARS, KNN, and ANN. It could be seen that the AUC of XGBoost, BT, and Mars were all 0.8, which means good prediction effects. Then, we further compared multiple machine learning algorithms and found that XGBoost had the best accuracy value (0.773) and kappa value (0.358) (Supplementary Figure 1). Therefore, XGBoost was selected as the machine learning model for predicting OA.

After determining the XGBoost model, we conducted a variable importance analysis. It could be seen that the top six important risk factors were age, BMI, Pb, hypertension, Mn, and Cd, respectively (Supplementary Figure 2).

In order to quantify the prediction effect of XGBoost model, we conducted confusion matrix analysis and reported the indicators of the model. The parameters of XGBoost were as follow: number of boosting trees = 1,000, max depth of trees = 10, eta= 0.001 and 0.05, gamma = 0.01, col sample by tree = 0.5, minimal child weight =1, subsample = 0.5.

**Figure 3:**
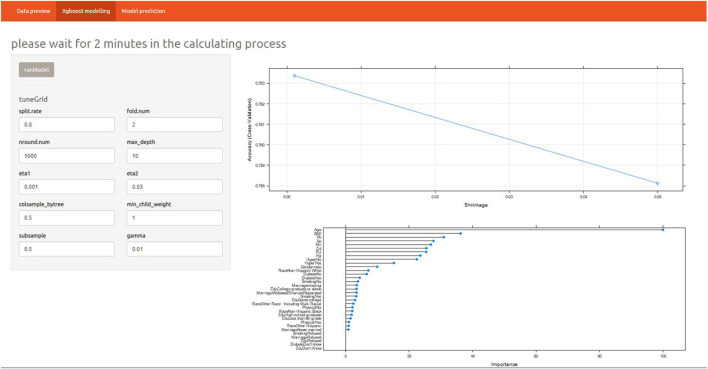
Introduction of online shiny in XGBoost model prediction.

The results included sensitivity 0.91, specificity 0.43, positive prediction value 0.8, negative prediction value 0.63, prevalence 0.72, detection rate 0.66, detection prevalence 0.81 and balanced accuracy 0.67 (Supplementary Table 1). Overall, the model had high sensitivity and good positive predictive value, while specificity and negative predictive value needed to be improved. To better take advantage of the prediction model, an online shiny application was developed (Figure 3). The details of the application were as below: https://alanwu.shinyapps.io/NHANES-OA/.

## Discussion

Based on the data of NHANES 2011-2022, this study explored the metal affecting the prevalence of OA. In the process of logistic regression fitting, individual information, living habits and disease history affecting OA were included. Finally, harmful risk factors were identified including Pb and Cd, and the protective factor was Mn. Neither Hg nor Se had a statistically significant effect on OA. In the further gender subgroup analysis, we obtained results consistent with overall logistic regression. Pb was a more obvious harmful factor of OA in men, while Cd was a significant risk factor in both men and women. Mn was not statistically significant in two different genders. The results of this study were consistent with many previous research reports (1, 18, 19).

Some studies analyzed the relationship between blood Lead and knee osteoarthritis and found that blood Lead were associated with the presence and severity of knee osteoarthritis (20). They proposed a potential mechanism: Pb increased turnover of bone, mineralized cartilage and non-mineralized cartilage. Pb exposure delayed fracture healing and inhibited the progress of endochondral ossification, which was related to bone remodeling in the development of OA. In addition, Pb exposure might further promote OA by increasing oxidative stress, which was the result of the increase of reactive oxygen species and the decrease of nitric oxide availability (21). In terms of blood Pb and personal information, the study also reported that women with high blood Pb tended to have higher BMI than men. For example, in the birth cohort, blood Pb increased in 1–3-year-old children, BMI would increase by 0.37 kg / m^2^ at the age of 17. Chinese women had the highest blood Pb content in one quarter (≥ 62.17 μg/L compared with women in the lowest quartile of blood). The increase of BMI would undoubtedly increase the prevalence of osteoarthritis (1).

Heavy metal cadmium (Cd) was an environmental pollutant, which could result in kidney injury and bone loss. After Cd entered the human body, the half-life of Cd was very long, about 10–30 years. Therefore, even under low-dose exposure, the long-term accumulation of cadmium would make it potentially toxic. Cd could promote the expression of enzymes related to the degradation of extracellular matrix in articular cartilage, such as metalloproteinases (MMP1, MMP3, MMP9 and MMP13), affecting COL II and aggrecan. IL-1 and IL-6 were associated with inflammatory responses and reduced the presence of glycosaminoglycans with proteoglycans by producing reactive oxygen species (19). At the same time, the pathogenesis of OA might be related to Cd induced chondrocyte injury. In addition, after Cd exposure, the high expression of metallothionein might reduce the sensitivity of OA to Cd (22). In Cd induced osteoporosis, osteoblasts might maintain their normal activity despite abnormal bone formation of osteoblasts and bone resorption of osteoclasts (17). In conclusion, the dysfunction of osteoblasts and osteoclasts might be the main cause of Cd induced osteoporosis.

By contrary, Manganese could slow down the degeneration of articular cartilage and was conducive to the repair of articular cartilage. Das et al. reported that the combined use of manganese, glucosamine, and chondroitin could regulate the metabolism of articular cartilage matrix, alleviate OA symptoms and improve imaging indicators (23). Manganese deficiency impaired glycosaminoglycan biosynthesis, leading to cartilage dysplasia characterized by OA and deformity (18).

As for metallic mercury, previous studies suggested that mercury was closely related to the occurrence of osteoarthritis and they found that Hg levels in the anterior cruciate ligament significantly increased in women under the age of 65 with spinal degenerative diseases (24). Hg in bones might be related to BMI, anatomical differences and gender. In this study, the correlation of Hg and OA was not confirmed, which might be due to the limitation of cross-sectional study design.

Finally, it was reported that several aspects of the relationship between Se and osteoarthritis remained controversial (25). Firstly, there was no difference in Se levels between OA and normal tissues. Secondly, the beneficial effect of Se supplementation on alleviating OA symptoms was uncertain (26).

Smoking was proved a risk factor for OA, and we found several interesting results in the further analysis. The smoking subgroup results showed that smoking might alleviate the association between Pb and OA a little, but might accelerate the correlation between Cd and OA. Non-smoking might obviously reduce the association between Pb and OA as well as the correlation between Cd and OA. The results of Ho study showed that smoking and lead exposure had a synergistic effect, which increased the oxidative stress in the human body (27). People with some gene variants might be sensitive to Pb and smoking through oxidative stress, suggesting that smoking cessation was an important issue in the Pb-exposed working environment. This conclusion was consistent with our results.

It was reported by Torres et al., that at the skeletal level, cadmium inhaled through tobacco smoke affected bone mineral density, leading to osteoporosis mediated by a reduction in antioxidant enzymes, which favor the process of bone resorption (28). In rheumatoid arthritis, tobacco use promoted citrullination through cadmium exposure and increases oxidative stress and inflammation, which coincided with our results.

Since machine learning algorithms were widely used in OA diagnosing and prediction (29–32), we tried to make a prediction of OA. Based on the three significant heavy metals Pb, Cd, and Mn in this study, we used a machine learning algorithm suitable for large-scale data, including 15,234 samples, including more than 10 other variables such as personal basic information, disease information and living habits. The XGBoost model with AUC of 0.8, accuracy value of 0.773 and kappa value of 0.358 was trained. The model could be used to predict OA for individuals, and provided a theoretical basis for further OA targeted interventions. The performance of XGBoost was confirmed by Lu et al. (33), who stated that XGBoost model with 15 variables had a high potential to predict venous thrombosis risk in patients with OA. Furthermore, we constructed an online prediction software for OA, which might be helpful for understanding the machine learning model and application of the prediction.

However, there were several limitations of our search. Firstly, the data used in this study were cross-sectional design, so it was impossible to infer the causal relationship between heavy metals and OA. Secondly, although we adjusted some demographic, medical history and lifestyle factors in logistic regression, there were still some confounding variables that may affect the correlation between metals and OA, but these variables were not considered. Thirdly, the blood samples of the participants were collected and measured at one time, and the single point measurement of metal might not reflect continuous exposure. Fourthly, the lack of information among participants might lead to the exclusion of several relevant results. The last limitation was the self-report of OA evaluation. The strengths of this research were as follow: Firstly, this research was based on big data (*N* = 15,234) from 2011 to 2020. Secondly, we made further subgroup analysis for the association between heavy metals and OA. Thirdly, machine learning algorithm was conducted for OA prediction involving a big data input to guarantee a good accuracy.

## Conclusion

In conclusion, we identified three heavy metals including Pb and Cd aggravated the prevalence of OA while Mn could be a protective factor of OA among the US aging population. Furthermore, we used online XGBoost model to make the prediction of probability in OA when demographic, disease history, lifestyle and heavy metal data were put into the model.

## Data availability statement

The datasets presented in this study can be found in online repositories. The names of the repository/repositories and accession number(s) can be found in the article/Supplementary material.

## Author contributions

JW: conception and design. XL: administrative support. QL: provision of study materials or patients. FX: collection and assembly of data, data analysis, and interpretation. All authors wrote the manuscript and approved final manuscript. All authors contributed to the article and approved the submitted version.

## Conflict of interest

The authors declare that the research was conducted in the absence of any commercial or financial relationships that could be construed as a potential conflict of interest.

## Publisher's note

All claims expressed in this article are solely those of the authors and do not necessarily represent those of their affiliated organizations, or those of the publisher, the editors and the reviewers. Any product that may be evaluated in this article, or claim that may be made by its manufacturer, is not guaranteed or endorsed by the publisher.

## Supplementary material

Supplementary Figure 1 Comparison among eight machine learning algorithms in accuracy and kappa values.

Supplementary Figure 2 Variable importance plot of OA prediction XGBoost model.

Supplementary Table 1 Confusion matrix of XGBoost model of OA prediction.

## Abbreviations

NHANES: National Health and Nutrition Examination Survey
BMI: body mass index
OR: odds ratio
CI: confidence interval
AUC: Area under the curve
ROC: Receiver Operating Characteristic
RF: Random Forest
SVM: Support vector machine
DT: Decision Tree
BT: Boosted Tree
MARS: Multivariate Adaptive Regression Splines
KNN: K-nearest neighbors
ANN: Artificial neural network
OA: Osteoarthritis
Pb: Lead
Hg: mercury
Cd: cadmium
Mn: manganese
Se: selenium
GM: geometric mean
PLI: poverty level index
DALY: disability adjusted life year
LOD: limit of detection
MMP1: Matrix Metallopeptidase 1
MMP3: Matrix Metallopeptidase 3
MMP9: Matrix Metallopeptidase 9
MMP13: Matrix Metallopeptidase 13
COL II: Collagen Type II
IL-1: Interleukin-1
IL-6: Interleukin-6.
