# Supplementary figures and images for: Identification for heavy metals exposure on osteoarthritis among aging people and Machine learning for prediction: A study based on NHANES 2011-2020
Source: Front Public Health. 2022 Aug 1;10:906774. doi: 10.3389/fpubh.2022.906774 (PMC9376265; doi:10.3389/fpubh.2022.906774)

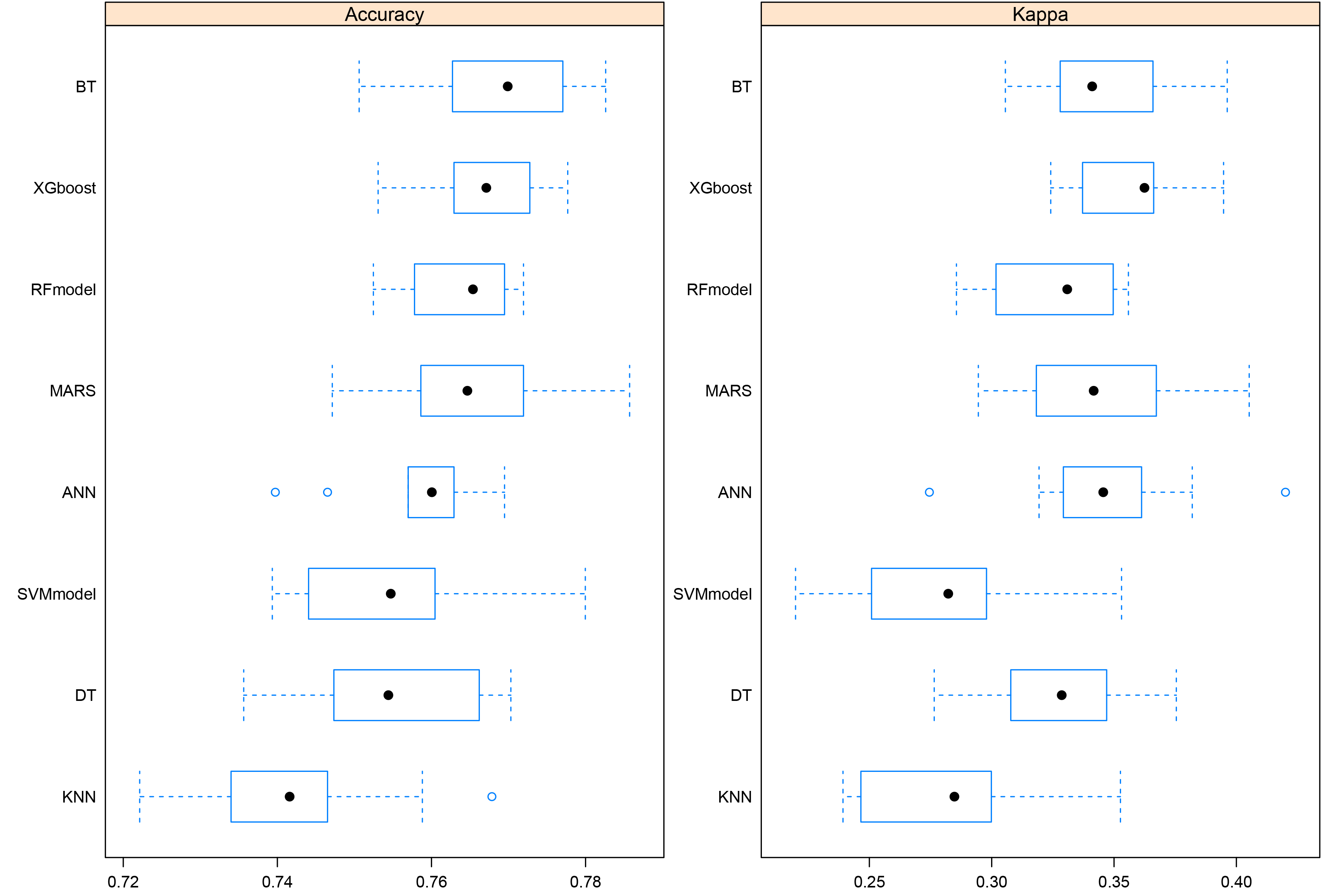

Supplement: Supplementary Figure 1 — Comparison among eight machine learning algorithms in accuracy and kappa values. [file Image_1.TIF]

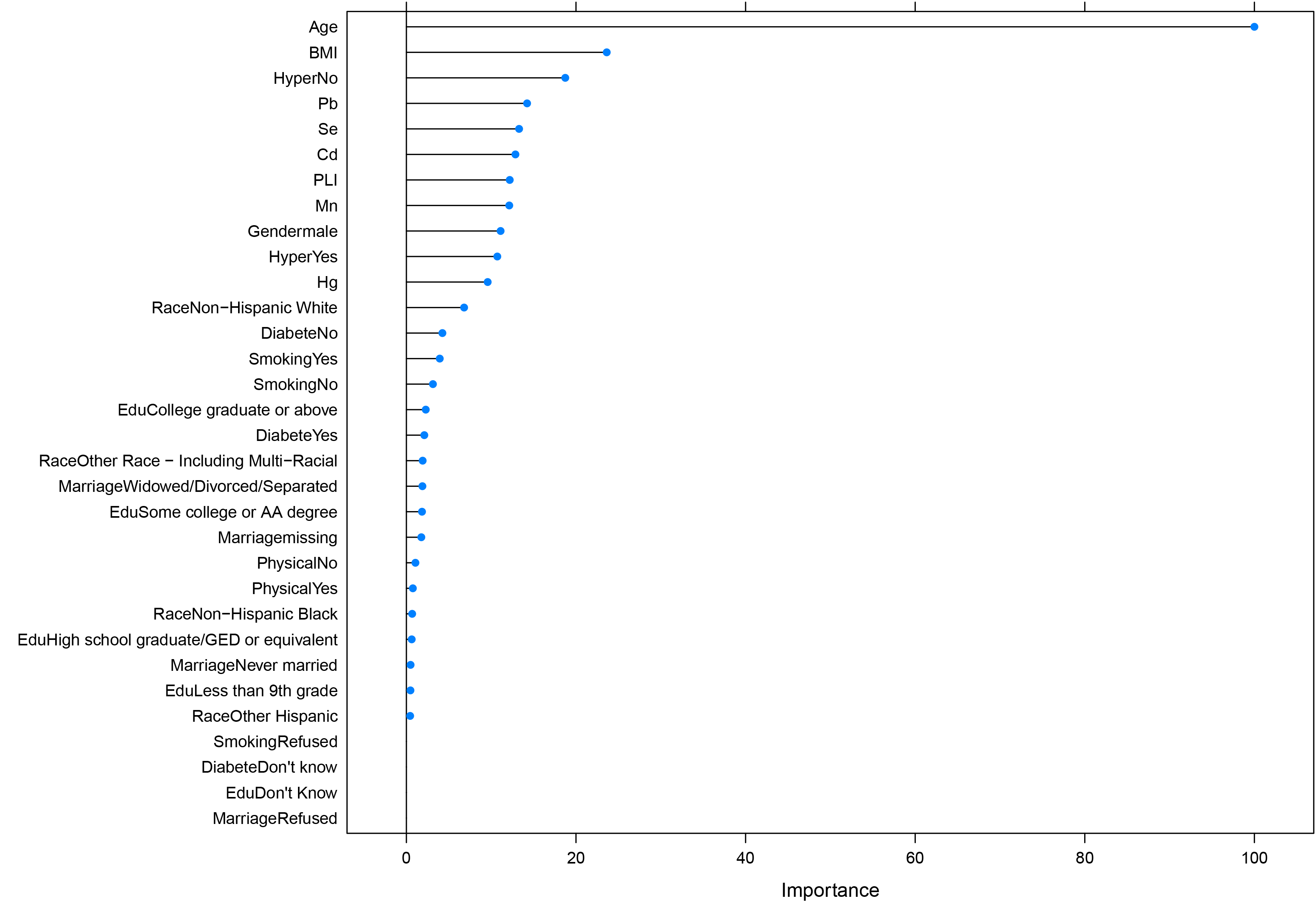

Supplement: Supplementary Figure 2 — Variable importance plot of OA prediction XGBoost model. [file Image_2.TIF]
