# Supplementary material for: Identification for heavy metals exposure on osteoarthritis among aging people and Machine learning for prediction: A study based on NHANES 2011-2020
Source: Front Public Health. 2022 Aug 1;10:906774. doi: 10.3389/fpubh.2022.906774 (PMC9376265; doi:10.3389/fpubh.2022.906774)
Supplement: Supplementary Table 1 — Confusion matrix of XGBoost model of OA prediction. [file Table_1.DOCX]

**Supplementary Table 1.** Confusion matrix of XGboost model of OA prediction

| Item | Value |
| --- | --- |
| Sensitivity | 0.91 |
| Specificity | 0.43 |
| PosPred Value | 0.8 |
| NegPred Value | 0.63 |
| Prevalence | 0.72 |
| Detection Rate | 0.66 |
| Detection Prevalence | 0.81 |
| Balanced Accuracy | 0.67 |
